# An Alternative Treatment Strategy for Complicated Chronic Wounds: Negative Pressure Therapy over Mesh Skin Graft

**DOI:** 10.1155/2017/8395219

**Published:** 2017-02-19

**Authors:** Michele Maruccia, Maria G. Onesti, Valentina Sorvillo, Antonio Albano, Luca A. Dessy, Bruno Carlesimo, Mauro Tarallo, Marco Marcasciano, Giuseppe Giudice, Emanuele Cigna, Diego Ribuffo

**Affiliations:** ^1^“Valdoni” Surgery Department, Plastic and Reconstructive Surgery Unit, “Sapienza” University of Rome, Viale del Policlinico 155, 00161 Roma, Italy; ^2^Emergency and Organ Transplantation Department, University of Bari “Aldo Moro”, Plastic and Reconstructive Surgery and Burns Unit, Piazza Giulio Cesare 11, 70124 Bari, Italy

## Abstract

Extensive skin defect represents a real problem and major challenge in plastic and reconstructive surgery. On one hand, skin grafts offer a practical method to deal with skin defects despite their unsuitability for several complicated wounds. On the other hand, negative pressure wound therapy (NPWT), applied before skin grafting, promotes granulation tissue growth. The aim of the study is to evaluate the improvement in wound healing given by the merger of these two different approaches. We treated 23 patients for large wounds of multiple factors. Of these, 15 were treated with the application of V.A.C.® Therapy (KCI Medical S.r.l., Milan, Italy), in combination with skin grafts after a prior unsuccessful treatment of 4 weeks with mesh skin grafts and dressings. Another 8 were treated with only mesh skin graft. Pain reduction and wound area reduction were found statistically significant (*p* < 0.0009, *p* < 0.0001). Infection was resolved in almost all patients. According to our study, the use of the negative pressure wound therapy over mesh skin grafts is significantly effective especially in wounds resistant to conventional therapies, thereby improving the rate of skin graft take.

## 1. Introduction

Chronic wounds, which typically result from medical conditions, nutritional deficiencies, infections, and metabolic disorders, involve intensive and time-consuming treatments [1]. Extensive skin defect is a great challenge and a major problem in plastic and reconstructive surgery. The use of skin grafts, whether split thickness or full thickness, has offered a reasonable method to address this problem. However the sole use of skin autologous grafts in complicated wounds might be not entirely satisfactory [1].

Negative pressure wound therapy (NPWT) is a therapeutic technique, which facilitates the healing of acute and chronic wounds [1]. NPWT, which deters the accumulation of fluid at wound sites through continuous drainage, makes daily dressing changes unnecessary, improves regional blood flow, and reduces bacterial proliferation, thus reducing the chances of infection [1]. NPWT can be successfully used before skin grafting, promoting granulation tissue growth with a 54% increase in growth rate and improving the survival rate of free skin graft [2–6].

The aim of the study is to evaluate the improvement in wound healing given by the merger of these two different approaches. Thereby the study underlines the crucial role of NPWT applied not only before but also on top of skin grafts. Our clinical study compares application of V.A.C. Therapy (KCI Medical S.r.l, Milan, Italy) in combination with skin grafts to repair acute and chronic wounds with the treatment of the same wounds covered only by a conventional skin graft.

## 2. Material/Patients and Methods

The study took place from January 2014 to June 2015 in the Department of Plastic and Reconstructive Surgery of “Sapienza” University of Rome.

A group of 23 patients were treated for large wounds of multiple factors. Of these 15 were treated with the application of V.A.C. Therapy (KCI Medical S.r.l, Milan, Italy), in combination with skin grafts after a prior unsuccessful treatment of 4 weeks with mesh skin grafts and dressing. Another 8 were treated with the only mesh skin graft. Inclusion criteria were wound area > 70 cm^2^. Exclusion criteria were patients with diabetes mellitus, patients treated with corticosteroids, and uncollaborative patients. Treatment was carried out on all by the same team.

A prospective-comparative study between mesh skin graft (SG) and mesh skin graft plus NPWT (SG+) was carried out evaluating several objective and subjective data before and after treatments on the same patient. The data collected before the first and the second treatment were disease duration, presence of pain, and wound characteristics such as size, exudation, and pain. In addition, a bacterial culture and sensitivity were performed for all the patients at the beginning of the treatment; after two weeks of SG; after 4 weeks; at the end of the first phase; and after two weeks of SG+. Before surgery, photos were made for every candidate.

**Figure 1:**
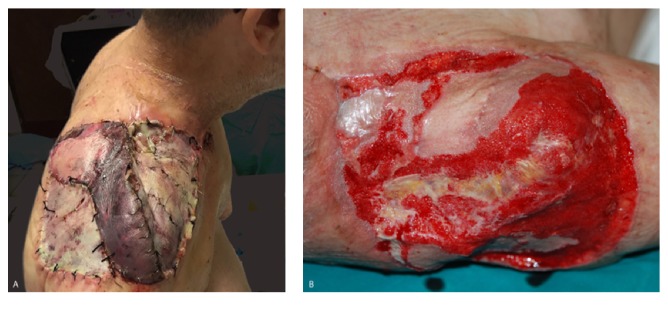
50-year-old patient affected by shoulder sarcoma after tumor excision and reconstruction with pedicled latissimus dorsi flap and skin grafts. (A) Seven days after surgery skin grafts and superficial latissimus dorsi flap necrosis due to* Pseudomonas aeruginosa* infection. (B) Area 21 days after surgical debridement and NPWT. Cultural examinations of wound continued to be positive for* Pseudomonas aeruginosa*.

**Figure 2:**
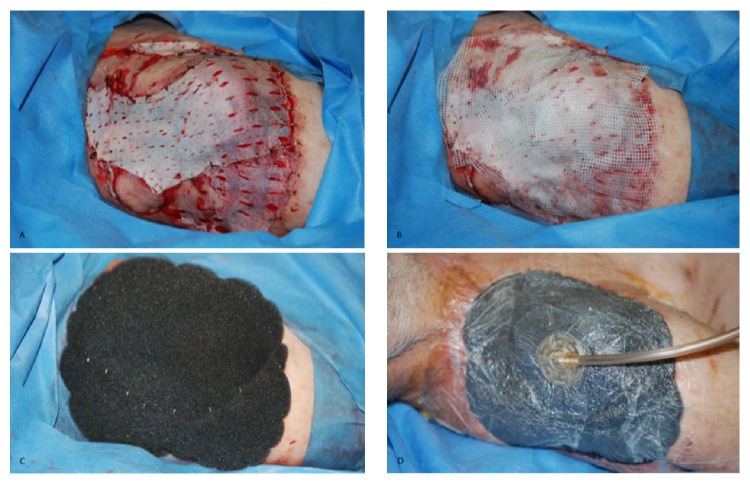
Surgical time. (A) Mesh skin grafts were placed over the wound and fixed; (B) placement of wet gauzes; (C) placement of NPWT; (D) negative pressure activation at −75 mmHg.

**Figure 3:**
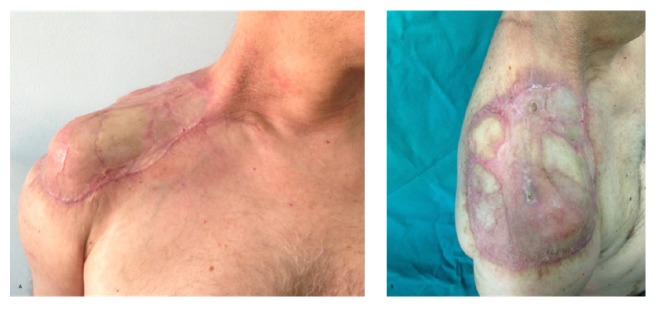
Postoperative results. (A) Anterior view 3 months after surgery; (B) lateral view 3 months after surgery with no signs of infection.

**Figure 4:**
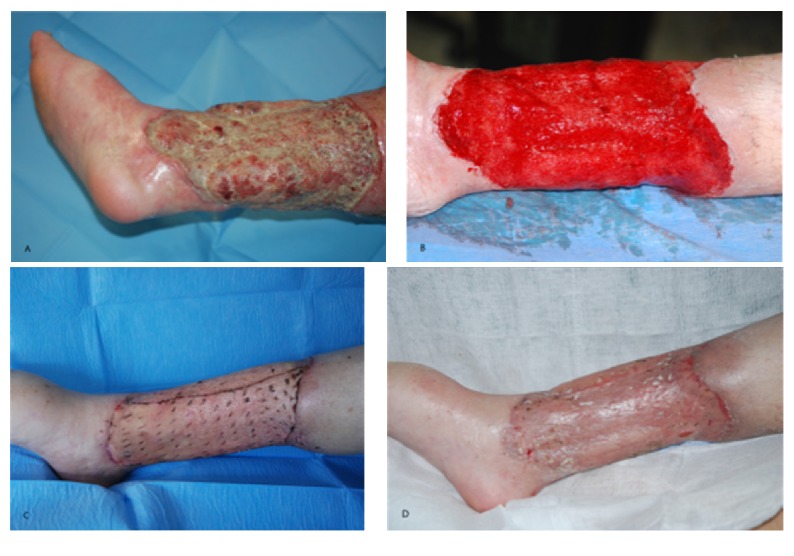
45-year-old patient affected by chronic infected lower limb ulcer. (A) Recovery time (cultural examinations were positive for* Pseudomonas aeruginosa* and* Enterococcus faecalis*); (B) after surgical debridement and antibiotic therapy (cultural examination was positive for* Pseudomonas aeruginosa*); (C) 7 days after meshed skin graft with NPWT; (D) 1 month after surgery.

**Figure 5:**
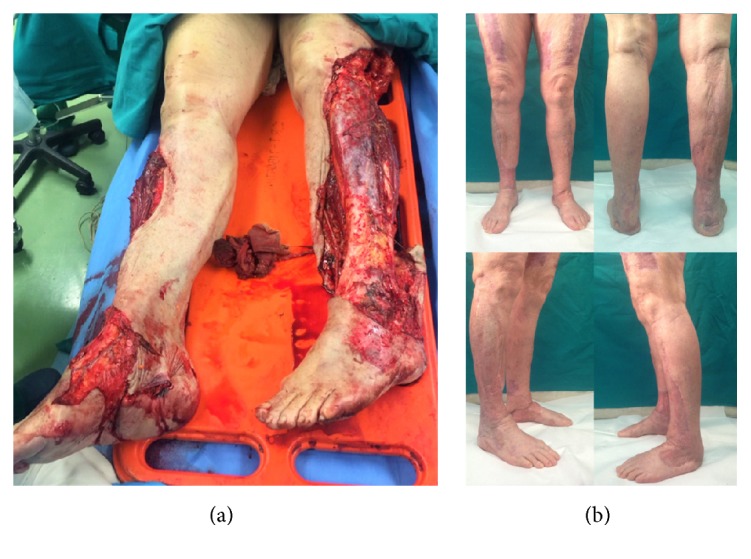
(a) 73-year-old patient after road accident with tram. She presented bilateral skin degloving of the lower limb. The patients underwent reconstruction with mesh skin grafts in traditional way for three times. This technique failed due to infection so the patient underwent reconstruction with our innovative protocol (mesh skin grafts + NPWT): (a) recovery time; (b) 1-year follow-up.

All patients underwent the same surgical protocol: in the first period, a mesh skin graft was placed and treated in traditional way (wet gauze application and compression). Whenever mesh skin grafts failed (4 weeks after surgery) the innovative surgical protocol was applied: wound disinfection with chlorine-based disinfectant and povidone-iodine, washing with saline solution, and application of mesh skin grafts. In addition NPWT was placed over the wet gauze for compression and immobilization. NPWT was set at −75 mmHg of aspiration. After five days the dressing was changed for a first evaluation of engraftment. NPWT was in place until grafts were engrafted and no more exudation was present (Figures 1–5(b)).

After 4 weeks of treatment (either SG and SG+) results were evaluated taking into account wound area (assessed as an ellipsoid) [7], status of the grafts, cultural examination, and pain relief. In order to describe subjective pain sensation, visual analogic scale (VAS) was submitted to the patients at the start of the treatment and on a weekly basis during the 2-month study period. A range from 1 (no pain) to 10 (maximum pain level) was considered. Wound margins were evaluated for necrosis, dermatitis, or, on the contrary, full integrity; periwound skin was evaluated for erythema, edema, and full integrity. Pain relief and area reduction were compared and analyzed using Student's* t*-test in two groups.

## 3. Results

Of the 23 patients treated in this study, 14 were males and 9 were females. Epidemiological data showed an average of 52 years (between 19 and 87 years). Etiologies were manifold: 15 had chronic vascular wound and 8 posttraumatic ulcers. In the first 4 weeks of treatment, complete healing occurred in 8 patients.

Complete healing occurred in 14 patients over a period of 10 weeks (6 of SG+). Complete initial engraftment occurred in 12 patients over a period of 8 weeks (4 of SG+). Only 1 patient was partially responsive to both treatments. In any case a remarkable reduction in wound exudation, edema, and perilesional margins was registered. Pain reduction was significant during the first period in 8 patients (average reduction of 1 point in the first week and 2.3 points at the end of the fourth week), the same ones whose wounds healed at the end of the fourth week. Pain reduction was significant in the second period in all patients not healed (15), with an average reduction of 2.1 points in the first week and 4 after 6 weeks.

An interesting trend was highlighted in the 15 patients entered in the second stage of therapy. In fact in the first 4 weeks of treatment only an average pain reduction of 1.04 points was reported, quite insignificant in comparison with the 2.13-point reduction after the first week of SG+ treatment (*p* < 0.0047). Another interesting piece of data was the comparison between pain relief after the first phase (mean = 3.39) and the second phase (mean = 6.13) with a *p* < 0.0009 (Tables 1 and 2).

Taking into account the second analyzed parameter, wound area reduction, significant results were found (*p* < 0.0001, *t* = 4.66); a mean reduction of 40.5% was found in the first period in comparison with 96.3% in the second period (Tables 3 and 4).

Cultural examinations of wound exudates at time 0 showed 8 patients positive for* Staphylococcus aureus*, 2 for* Pseudomonas aeruginosa*, and 1 for* Enterococcus faecalis*. After two weeks of SG, 7 patients were positive for* Staphylococcus aureus*, 2 for* Pseudomonas aeruginosa*, and 1 for* Enterococcus faecalis*. After 4 weeks* Staphylococcus aureus* was present in 5 patients,* Pseudomonas aeruginosa* in 2 patients, and* Enterococcus faecalis* in 1 patient. Cultural examinations were repeated after 2 weeks of SG+ and shown the presence of* Staphylococcus aureus* in 2 patients and* Pseudomonas aeruginosa* in 1 patient (Table 5).

## 4. Discussion

Chronic ulcer usually means an injury neither spontaneously evolving towards healing nor progressing through normal stages of healing such as inflammation, proliferation, and remodeling. This condition represents a serious physical and psychological limit to the patient as well as being a burden for the health system [7].

Treatments in patients with chronic wounds usually consist in debridement and skin grafts. An important innovation in chronic wounds management is the use of NPWT over the skin graft. Our advice would be a trick we developed: use 75 mmHg negative pressure on the skin graft instead of 125 mmHg. In our experience this is very important in order to allow good adhesion of the skin graft to the wound bed during initial engraftment. We evaluated the data reported and highlighted a marked and statistically significant improvement in the treatment of large loss of substance through mesh skin grafts dressed with V.A.C. Therapy system (*p* < 0.001). In fact, taking into account the 23 patients enrolled in the study, it was pointed out that only 8 of them healed through traditional treatment, while the remaining 14 out of 15 healed with the modified treatment in 6 weeks. These data provide us with various interpretations: the 15 patients who remained in the study were those who had a wound whose healing was trickier. It is also interesting to evaluate how the data “pain relief” strongly and quickly improved (in a statistically significant way) already after the first week of SG+ treatment in the group of patients who had reported only a marginal improvement in the first phase of the study.

Another important fact to consider is the presence of infection in the examination cultures after 4 weeks (end of the first phase of treatment) in comparison with the examination carried out after 6 weeks. In fact after 4 weeks 5 people reported positive culture examination for* S. aureus* infection, 2* P. aeruginosa*, and 1* E. faecalis*; after only 2 weeks of treatment with SG+NPWT only 2 people still had positive wound swab for* S. aureus* and 1* P. aeruginosa*. We observed also an improvement of the other signs and symptoms of infection as purulent discharge from the wound, pain, swelling, and redness. With the data available we can say that the use of NPWT above mesh skin grafts is significantly better than the mesh skin grafts alone. In addition it showed a faster recovery and a better dressing management and a reduced number of dressing changes. Numerous studies have been carried out on the combined use of NPWT over dermal substitutes also to cover large and deep wounds (including bone and tendon exposure) [7–13]. Every study assessed that, thanks to the integrated use of these techniques, the preparation of the wound bed was quicker in order to obtain a better graft take [14]. In fact NPWT used over the dermal template delivered a faster maturation (approximately 1-2 weeks) and a higher integration quality [15, 16]. This integrated technique keeps the matrix immobilized, leaves the wound bed moist and free of debris, prevents the accumulation of fluid collections, and reduces bacterial colonization of the wound [13]. Likewise biological semipermeable membranes, connected through drainage tubes to vacuum source, create a fully closed negative pressure drainage condition promoting the expulsion of effusion and liquefied necrotic tissue in the cavities [17]. This kind of dressing results in a better immobilization of the graft, thereby limiting shear forces, eliminating fluid collections, bridging of the graft, and decreasing bacterial contamination.

## 5. Conclusion

According to our study the use of the negative pressure wound therapy over mesh skin grafts is dramatically effective especially in wounds resistant to conventional therapies. Although there are no current studies on the two techniques combined, our data are comparable to those on the integration of dermal substitutes and NPWT.

## Figures and Tables

**Table 1:** Pain relief in patients during 10 weeks valued with VAS.

Patient	Time 0	1 week	4 weeks	5 weeks	6 weeks	Pain
1	7	−3	−4	—	—	0
2	6	−2	−4	—	—	0
3	7	−1	−6	—	—	0
4	8	−2	−6	—	—	0
5	6	−3	−3	—	—	0
6	7	−1	−6	—	—	0
7	8	−3	−5	—	—	0
8	6	−3	−3	—	—	0
9	7	−1	−1	−1	−4	0
10	8	+1	−1	−3	−5	0
11	8	−1	−1	−1	−5	0
12	8	−1	−1	−2	−4	0
13	7	−1	−1	−2	−3	0
14	7	0	−1	−3	−5	0
15	6	0	−2	−1	−3	0
16	8	−1	−2	−3	−2	0
17	8	0	−1	−3	−4	0
18	7	−1	−1	−2	−3	0
19	8	−2	−1	−1	−4	0
20	7	+1	−2	−2	−4	0
21	9	0	0	−3	−5	5
22	8	0	−1	−2	−5	0
23	6	0	−1	−3	−4	0
*Mean*	*7.26*	*−1.04*	*−2.3*	*−2.1*	*−4*	

**Table:** (a) Comparison of pain relief in week 1 and week 5

*N*	M	*p*	95% ci	df	sed	*t*
23 (SG) 15 (SG+)	1.04 2.13	<0.0047	−1.82/−0.36	36	0.361	3.015

**Table:** (b) Comparison of pain relief in week 4 and week 10

*N*	M	*p*	95% ci	df	sed	*t*
23 (SG) 15 (SG+)	2.35 4.0	<0.0035	−2.72/−0.58	36	0.528	3.127

**Table:** (c) Comparison of pain relief in first treatment (SG) and second treatment (SG+)

*N*	M	*p*	95% ci	df	sed	*t*
23 (SG) 15 (SG+)	3.39 6.13	<0.0009	−4.29/−1.2	36	0.761	3.6

**Table 3:** Comparison between area reduction (AR) in two phases of the study (SG, SG+).

Patient	AR (SG)	AR (SG+)
1	100	—
2	100	—
3	100	—
4	100	—
5	100	—
6	100	—
7	100	—
8	100	—
9	6	100
10	9	100
11	20	100
12	5	100
13	6	100
14	0	100
15	13	100
16	19	100
17	5	100
18	4	100
19	15	100
20	7	100
21	0	45
22	17	100
23	6	100
*Mean*	*40.5*	*96.3*

**Table 4:** Statistical analysis of the area reduction values.

*N*	M	*p*	95% ci	df	sed	*t*
23 (SG) 15 (SG+)	40.5 96.3	<0.0001	−80.08/−31.54	36	11.969	4.66

*N* = number of patients, M = mean, *p* = *p* value, 95% ci = 95% confidence interval, df = degree of freedom, sed = standard error of difference, and *t* = *t* value.

**Table 5:** Cultural examination, time 0, after 2 weeks, after 4 weeks, and after 6 weeks.

Patient	Time 0	2 weeks	4 weeks	6 weeks
1	—	—	—	—
2	—	—	—	—
3	SA	SA	—	—
4	SA	—	—	—
5	—	—	—	—
6	SA	SA	—	—
7	—	—	—	—
8	—	—	—	—
9	—	SA	SA	—
10	—	—	—	—
11	SA	—	—	—
12	EF	EF	EF	—
13	—	SA	SA	—
14	SA	SA	SA	SA
15	—	—	—	—
16	SA	—	—	—
17	—	—	—	—
18	PA	PA	PA	—
19	SA	SA	SA	SA
20	—	—	—	—
21	PA	PA	PA	PA
22	—	—	—	—
23	SA	SA	SA	—

SA = *Staphylococcus aureus*; PA = *Pseudomonas aeruginosa*; EF = *Enterococcus faecalis*.
